# A Systematic Review Assessing the Impact of Vitamin D Levels on Adult Patients with Lymphoid Malignancies

**DOI:** 10.3390/curroncol30040331

**Published:** 2023-04-20

**Authors:** Cristina Potre, Ema Borsi, Ovidiu Potre, Ioana Ionita, Miruna Samfireag, Dan Costachescu, Cristina Secosan, Sandra Lazar, Anca Irina Ristescu

**Affiliations:** 1 Department of Internal Medicine, Discipline of Hematology, “Victor Babes” University of Medicine and Pharmacy Timisoara, Eftimie Murgu Square 2, 300041 Timisoara, Romania; potre.cristina@umft.ro (C.P.); potre.ovidiu@umft.ro (O.P.); ionita.ioana@umft.ro (I.I.); 2 Department of Internal Medicine, Discipline of Clinical Practical Skills, “Victor Babes” University of Medicine and Pharmacy Timisoara, Eftimie Murgu Square 2, 300041 Timisoara, Romania; samfireag.miruna@umft.ro; 3 Department of Orthopedics-Traumatology, Urology, Radiology, and Medical Imaging, “Victor Babes” University of Medicine and Pharmacy Timisoara, Eftimie Murgu Square 2, 300041 Timisoara, Romania; costachescu.dan@umft.ro; 4 Department of Obstetrics and Gynecology, Discipline of Obstetrics and Gynecology, “Victor Babes” University of Medicine and Pharmacy Timisoara, Eftimie Murgu Square 2, 300041 Timisoara, Romania; secosan.cristina@umft.ro; 5 Second Department of Internal Medicine, “Victor Babes” University of Medicine and Pharmacy Timisoara, Eftimie Murgu Square 2, 300041 Timisoara, Romania; sandra.lazar@umft.ro; 6 Discipline of Anesthesia and Intensive Care, School of Medicine, “Grigore T. Popa” University of Medicine and Pharmacy, University Street 16, 700115 Iasi, Romania; 7 Department of Anesthesia and Intensive Care, Regional Institute of Oncology, General Henri Mathias Berthelot 2-4, 700483 Iasi, Romania

**Keywords:** vitamin D, survival analysis, substance addiction, hematologic malignancy, lymphoid tissue

## Abstract

Vitamin D deficiency has been correlated with various conditions, including the risk of developing lymphoid malignancies. This systematic review aimed to assess the association between vitamin D levels at diagnosis of lymphoid malignancies, patient outcomes, and survival. A systematic review was conducted, encompassing 15 studies published until January 2023, involving 4503 patients, examining the relationship between vitamin D and lymphoid cancers. The median age of the patients was 56.5 years, with a median follow-up duration of approximately 36 months across studies. The overall median vitamin D level at initial measurement was 20.4 ng/mL, while a <20 ng/mL threshold was used to define vitamin D insufficiency. The results demonstrated significant associations between vitamin D levels and patient outcomes in several lymphoid malignancies, with a pooled risk in disease progression of 1.93 and a pooled hazard ratio of 2.06 for overall survival in patients with 25-(OH)D levels below the normal threshold of 20 ng/mL. Among findings, it was demonstrated that supplemental vitamin D improves the chemosensitivity of tumors by reducing the rate of tumor growth compared with vitamin D or chemotherapy alone. Vitamin D had a protective effect for patients with DLBCL under R-CHOP treatment, while vitamin D insufficiency was associated with the impairment of rituximab treatment and showed worse clinical outcomes in chimeric antigen receptor T-cell (CAR-T) recipients. Although one study found no association between vitamin D deficiency and the cause of death, most associated vitamin D insufficiency with early clinical failure and lower survival probability. In conclusion, his systematic review highlights the importance of vitamin D levels in the prognosis and survival of patients with lymphoid malignancies. Further research is needed to better understand the underlying mechanisms and explore the potential benefits of vitamin D supplementation in managing these cancers.

## 1. Introduction

Vitamin D is a fat-soluble secosteroid hormone with essential functions for several physiological processes, including calcium homeostasis management, immunological modulation, and cell development [1,2]. The active form of vitamin D, 1,25-dihydroxy vitamin D (1,25(OH)2D), also known as calcitriol, exerts its effects through binding to the vitamin D receptor, a nuclear receptor found in several organs [3]. During activation, the vitamin D receptor forms a heterodimer with the retinoid X receptor, affecting the target genes in order to regulate their transcription [4], thus raising the hypothesis of the potential biological effects of vitamin D in cancer prevention and treatment [5,6,7].

Vitamin D’s antitumor capabilities stem from its capacity to control several cellular cancer-associated cellular processes, such as cellular proliferation and differentiation, apoptosis, angiogenesis, and metastasis of malignant cells [8,9]. First, calcitriol has been shown to inhibit the growth of several types of cancer cells by inducing cell cycle arrest through the upregulation of cyclin-dependent kinase inhibitors, such as p21 and p27, and the downregulation of cyclins, thereby inhibiting the progression of the cell cycle [10,11]. In addition, vitamin D promotes cellular differentiation, a process often dysregulated in cancer cells, as research studies suggest [12,13]. In numerous cancer cell lines, calcitriol has been demonstrated to increase the expression of differentiation markers and drive morphological changes indicative of differentiated cells, with a substantial impact on treating acute myeloid leukemia with vitamin D analogs [14,15].

Apoptosis, also known as programmed cell death, is a key mechanism of the body for eliminating potentially hazardous cells, such as malignant ones. One of the mechanisms behind calcitriol-induced apoptosis in many types of cancer cells has been observed via altering the expression of proapoptotic and antiapoptotic proteins, including B-cell leukemia/lymphoma 2 protein (BCL-2) family members and caspases [16,17]. Thus, apoptosis generated by vitamin D may contribute to its possible anticancer function and impede angiogenesis in developing tumors by inhibiting the production of proangiogenic molecules, such as vascular endothelial growth factor and matrix metalloproteinases [18,19]. Lastly, it has been shown that calcitriol exhibits anti-inflammatory effects, which may have an important significance for cancer prevention by inhibiting the production of proinflammatory cytokines and chemokines, which may contribute to its anticancer properties [20]. Therefore, it was hypothesized that vitamin D levels are significantly associated with the clinical outcomes, prognosis, and overall survival of adult patients with lymphoid malignancies.

In the current study, it was aimed to conduct a systematic review of the existing literature to evaluate the impact of vitamin D levels on the incidence, progression, and response to therapy in adult patients with lymphoid malignancies, such as non-Hodgkin lymphoma, Hodgkin lymphoma, and chronic lymphocytic leukemia. Another main aim was to assess the correlation between vitamin D levels and prognostic factors, including tumor stage, disease aggressiveness, and overall survival, in patients with lymphoid malignancies. The secondary aim of the study was to explore the potential role of vitamin D supplementation in the management of lymphoid malignancies, specifically its influence on treatment response, disease progression, and quality of life.

## 2. Materials and Methods

### 2.1. Review Protocol

This systematic review was conducted in February 2023, utilizing four online databases: PubMed, Web of Science, Cochrane, and Scopus. The review encompassed literature published up until January 2023. The investigation covered the following medical subject headings (MeSH) [21] keywords: “vitamin D”, “vitamin D deficiency”, “hematologic malignancies”, “hematologic malignancy”, “hematological neoplasms”, “neoplasia”, “cancer”, “malignancy”, “hematologic diseases”, “lymphoma”, “lymphocytic leukemia”, “lymphoid cells”, “lymphoid malignancy”, and “lymphoid tissue”. The search was restricted to English-language journal articles.

Employing a structured and systematic search strategy in compliance with the Preferred Reporting Items for Systematic Reviews and Meta-Analyses (PRISMA) [22] criteria and the International Prospective Register of Systematic Reviews (PROSPERO) [23] guidelines, all pertinent scientific papers examining the autopsy findings of the lungs in the elderly were incorporated into the analysis. This systematic review was registered on the Open Science Framework (OSF) platform [24].

The primary objective of this systematic review was to address the following research questions:

- *What are the vitamin D levels measured at diagnosis of lymphoid malignancies?*
- *Are there any significant differences in patient outcomes based on vitamin D levels?*
- *What is the influence of vitamin D on patient survival?*

### 2.2. Selection Process

The main sources of information for the gathered material included the text, tables, figures, and additional web resources present in the articles. The initial stage of the selection process involved the elimination of duplicate submissions, followed by a thorough examination of each abstract and, ultimately, a complete review of the entire text. Additionally, the reference lists of the collected papers were meticulously inspected to identify relevant content.

The criteria for including a study in the analysis were as follows: (1) the research should examine the association between vitamin D and lymphoid cancers, (2) the study must have measured the vitamin D levels at diagnosis, (3) the research must have detailed the outcomes and survival of these patients, (4) the study cohort must be older than 18 years. Conversely, the exclusion criteria were: (1) studies that did not measure vitamin D at diagnosis of a lymphoid malignancy; (2) studies lacking relevant data on patients’ characteristics and medical history; (3) studies including hematological malignancies other than lymphoid cancers; (4) studies examining children with hematological malignancies; (5) case reports, literature reviews, meta-analyses, letters to editors, and brief communications were also excluded from the selection.

In the context of our review, we considered the following variables to be considered for reporting in this review: (1) study characteristics: study number and author, country of the study, the year of study development, study design, and quality assessment; (2) summary of findings: number of patients, average/median age, the proportion of male/patients, types of lymphoma identified, outcome measure of the study, duration of follow-up; (3) evaluation of vitamin D levels: vitamin D threshold, vitamin D measuring method, mean/median vitamin D levels, the proportion of patients with mild/moderate/severe vitamin D insufficiency, the proportion of patients with optimal vitamin D levels; (4) outcomes evaluation: Newcastle–Ottawa scale, mortality, disease-free survival (DFS), overall survival (OS), and other study particularities.

### 2.3. Data Extraction and Quality Assessment

**Figure 1:**
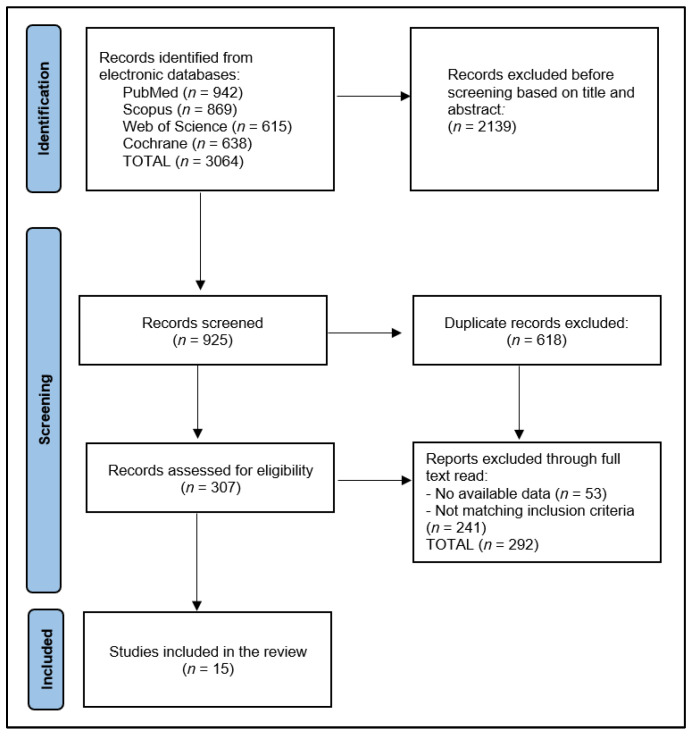
PRISMA flow diagram.

The preliminary search yielded 3064 articles, out of which 618 were identified as duplicates. After excluding 2139 papers based on their abstracts, 307 full-text articles were assessed for eligibility. Ultimately, 15 articles were selected for inclusion in the systematic review, as presented in Figure 1. Based on the Study Quality Assessment Tools provided by the National Heart, Lung, and Blood Institute (NHLBI) [24], two investigators independently evaluated the published material and documented their findings. These tools are tailored to specific study designs, enabling the detection of methodological or design concerns.

The Quality Assessment Tool for Observational Cohort and Cross-Sectional Investigations was employed for the remaining studies. Each question within the tool received a score of 1 point for “Yes” answers and 0 points for “No” and “Other” responses. Subsequently, the final performance score was calculated. Accordingly, studies with scores ranging from 0 to 4 were considered fair quality, those with scores between 5 and 9 were deemed good quality, and those with a score of 10 or higher were classified as excellent quality, as seen in Table 1. To mitigate inherent biases in the included studies, two researchers were assigned to evaluate the quality of the chosen articles. This approach minimized the risks associated with selection bias, missing data, and measurement bias.

## 3. Results

Overall, a total of 4503 patients were analyzed across the 15 studies included in this systematic review. A comprehensive summary of findings from the included studies is presented in Table 2, detailing key demographic and clinical characteristics of all patients described. The total number of patients in the studies ranged from 70 to 983, with study 1 [25] by Drake et al. including the largest sample size. Age was reported in most studies, with an overall median age of 56.5 years. The median age ranged from 32 to 67 years, while the study by Schanafelt et al. [26] had the highest median age (67 years), and the study by Borchmann et al. [34] had the lowest median age, with only 32 years.

Gender distribution expressed as a proportion of men varied from 35.8% in the study by Tretli et al. [27] to 86.6% in the study by Aref et al. [28]. The studies encompassed several cancer types, including diffuse large B-cell lymphoma (DLBCL), T-cell lymphoma (TCL), B-cell lymphoma (BCL), mantle cell lymphoma (MCL), follicular lymphoma (FL), splenic marginal zone lymphoma (MZL), extranodal MZL, nodal MZL, lymphoplasmacytic lymphoma (LPL), Burkitt lymphoma (BL), non-Hodgkin lymphoma (NHL), chronic lymphocytic leukemia (CLL), and extranodal natural killer/T-cell lymphoma (ENKTL). Among these cancer types, DLBCL emerged as the most frequently studied, featuring in six of the fifteen studies with a total of 1629 patients [25,29,31,35,38,39]; FL was analyzed in four other studies encompassing 1346 patients [25,30,31], BCL in three studies [25,28,33], while CLL [26], MCL [34], and ENKTL [36] were present in one study.

Outcome measures included overall survival (OS), disease-free survival (DFS), time to treatment (TTT), and complete response (CR). Most studies reported OS and DFS as the primary outcome measures. The duration of follow-up varied substantially among the studies, ranging from 20 months in the study by Djurasinovic et al. [31] to 156 months in Borchmann et al.’s study [34]. Overall, the median duration of follow-up across all studies was approximately 34.8 months.

Table 3 presents a detailed evaluation of vitamin D levels in adult patients with lymphoid malignancies from the included studies, comprising vitamin D threshold levels, the method of measurement, mean or median vitamin D levels, and prevalence of insufficiency among the studied groups. The majority of studies (7 out of 15) used a threshold of <20 ng/mL to define vitamin D insufficiency, while five studies applied a <30 ng/mL threshold [31,32,34,37,38], and one study adopted a <8 ng/mL threshold [29]. A variety of measuring methods were employed across the studies, including liquid chromatography–tandem mass spectrometry (LC-MS/MS), radioimmunoassay (RIA), enzyme-linked immunosorbent assay (ELISA), chemiluminescence immunoassay (CLIA), and electrochemiluminescence (ECL).

Mean or median vitamin D levels were reported in 12 out of the 15 studies, ranging from 9.2 ng/mL in the study by Bittenberg et al. [29] to 31.0 ng/mL in Kelly et al.’s study [30] and an overall median value at the initial measurement of 21.8 ng/mL. Severe insufficiency, defined as a serum vitamin D level lower than 10 ng/mL, was reported in six studies, with the highest percentages of patients in studies 6 [30], 12 [39], and 5 [29], respectively. Overall insufficiency rates were reported in all studies except for study 7 [31]. The highest overall insufficiency rate (92.8%) was found in study 14 [38], while the lowest rate (18.7%) was reported in study 7 [31]. Optimal vitamin D levels, defined as equal to or greater than the threshold value, were reported in all studies, with the highest percentage of patients having optimal levels observed in the Tracy et al. study [31] (81.3%) and the lowest in the study by Bittenberg et al., with only 0.3% [29].

Table 4 provides an evaluation of outcomes across 15 studies, examining the relationship between vitamin D levels and various aspects of patient survival and treatment response, the main outcomes being disease-free survival (DFS), overall survival (OS), time to treatment (TTT), and complete response to treatment (CR), described by the hazard ratio (HR) and odds ratio (OR) for patients with insufficient vitamin D levels. In the study by Drake et al. [25], the overall death rate was 19.6%, with 17.1% of deaths attributed to lymphoid cancer. The study found a significant association between vitamin D levels and survival for diffuse large B-cell lymphoma (DLBCL) and T-cell lymphoma (TCL), with significant hazard ratios for DFS and OS of 1.41 and 1.99, respectively. Schanafelt et al. [26] reported an overall death rate of 17.7% and observed a significant association between vitamin D levels and TTT (HR = 1.47), but not for OS.

In the study by Tretli et al. [27], there was a death rate of 51.7% from lymphoma during the average 72 months of follow-up and a significantly higher survival rate among patients with the highest vitamin D levels (HR = 3.03). Aref et al. [28] reported a death rate of 66.6% during the 60 months of follow-up and demonstrated that patients with normal vitamin D levels had a longer average survival (56.8 months) compared with those with deficient levels (48.7 months), with a 5.26 times higher risk of death. Bittenberg et al. [29] found that vitamin D deficiency impairs the effect of rituximab, with a death rate of 30.1% and a hazard ratio of 4.10 for overall survival. In contrast, study 6 by Kelly et al. [30] found no association between vitamin D deficiency and cause of death (10.4% death rate), with no significant DFS or OS. Study 7 by Tracy et al. [31] reported a death rate of 12.1% and found an association between vitamin D insufficiency and early clinical failure (OS HR = 2.35). Borchmann et al. [34] demonstrated that supplemental vitamin D improves the chemosensitivity of tumors by reducing the rate of tumor growth compared with vitamin D or chemotherapy alone, with DFS and OS HRs of 2.13 and 1.82, respectively.

Wang et al. [35] found strong evidence for a relationship between 25-(OH)D levels and prognosis in DLBCL and validated the link between vitamin D and c-Myc expression, with a death rate of 23.6%, DFS HR of 2.82, and OS HR of 3.72. In the studies by Xu et al. [36] and Mao et al. [37], it was reported that 25-(OH)D deficiency was a significant negative prognostic predictor for mantle cell lymphoma (MCL) and extranodal natural killer/T-cell lymphoma (ENKTL), with DFS and OS hazard ratios of 3.17 and 8.30, and 2.60 and 2.93, respectively. Chen et al. [38] found in study 12 that vitamin D had a protective effect for patients with DLBCL under R-CHOP treatment, with DFS and OS HRs of 1.58 and 2.50, respectively. Lastly, Nath et al. [39] demonstrated a link between insufficient vitamin D levels and worse clinical outcomes in chimeric antigen receptor T-cell (CAR-T) therapy recipients, with an HR of 2.58 for CR and 2.24 for overall survival.

## 4. Discussion

### 4.1. Summary and Contributions

The majority of studies suggest a significant association between insufficient vitamin D levels and negative outcomes for patients with lymphoid malignancies, including reduced survival and impaired treatment response. However, the relationship between vitamin D deficiency and patient outcomes appears to be more pronounced in certain subtypes of lymphoid malignancies, such as DLBCL, TCL, MCL, and ENKTL. Further, some studies indicate that vitamin D supplementation may improve treatment efficacy, including chemosensitivity and response to rituximab. Nonetheless, it is important to note that other studies, such as that by Kelly et al. [30], did not find a significant association between vitamin D deficiency and cause of death or survival outcomes. This discrepancy highlights the need for further research to corroborate the findings and explore the mechanisms underlying the relationship between vitamin D levels and lymphoid malignancies.

The observed associations between 25-hydroxyvitamin D (25(OH)D) levels and certain patient characteristics, such as poor performance status, low albumin levels, parameters of disease activity, and B-symptoms, can be attributed to factors such as reduced physical activity, sunlight exposure, and dietary vitamin D intake, as well as the role of the liver in 25(OH)D generation [40]. The underlying mechanism for the association between B-symptoms and vitamin D levels remains unclear; however, one study has identified a significant association between lower vitamin D levels and higher interleukin-6 (IL-6) levels [41]. Additionally, the inverse association between 25(OH)D and hemoglobin can be explained by recent data suggesting that vitamin D can modulate the expression of the iron regulatory peptide hepcidin, which is involved in the development of anemia of chronic inflammation. The activation of the IL-6/hepcidin axis was previously demonstrated in patients with Hodgkin and aggressive B-cell lymphomas [42]. In this study, the prognostic value of baseline 25(OH)D levels was confirmed on outcomes lymphomas, determining a significant association between low 25(OH)D levels (below 20 ng/mL) and significantly lower disease-free survival.

Threshold values for prognostic relevance varied between studies, potentially reflecting geographical or ethnic differences or the influence of factors such as climate, diet, or lifestyle habits. The results indicated that patients with lymphoproliferative disease and severe 25(OH)D deficiency before treatment had a significantly shorter time to progress than patients with higher serum levels, which supported earlier findings [28]. The study showed a higher frequency of severe 25(OH)D serum deficiency in patients with lymphoproliferative disease than previously reported and compared to the general population in Europe and the USA [43]. Patients with severe serum 25(OH)D deficiency had more frequent worse ECOG PS, and malnourished patients and those at risk of malnutrition were more likely to have severe serum 25(OH)D deficiency.

There is a plausible biological basis for the involvement of vitamin D in enhancing antibody-mediated antitumor macrophage activity, prompting the supplementation of vitamin D in patients with lymphomas [44]. In the study by Hohaus [33], a vitamin D supplementation regimen was devised, incorporating a loading phase based on the patient’s baseline serum 25(OH)D concentration and a maintenance dose of 3570 IU/day, which exceeds the recommended daily limit of 2000 IU/day [45]. However, a 10,000 IU/day threshold has been suggested as the level at which no adverse effects are observed in healthy individuals, and doses up to 10,000 IU/day have been deemed safe for breast cancer patients for up to 4 months since vitamin D toxicity typically occurs after approximately 40,000 IU/day of continuous use [46].

Incorporating an initial loading phase led to a more substantial increase in 25(OH)D levels after a median of 6 weeks compared with the weekly supplementation approach in the Hohaus et al. study [33]. However, nearly half of the patients still exhibited below normal 25(OH)D levels, indicating that those undergoing immunochemotherapy, including corticosteroid treatment, may require higher vitamin D intake. Consistent with these findings, more than 40% of lung cancer patients receiving a daily 20,000 IU vitamin D3 loading regimen did not achieve the target level within the expected time [46]. Additionally, a recent study reported weekly supplementation of 50,000 IU of vitamin D in lymphoma patients with vitamin D insufficiency. After 12 weeks, all but one patient attained normal vitamin D levels > 25 ng/mL [47].

In addition, there were few reports comparing serum 25(OH)D levels among subtypes of lymphomas, revealing a significant percentage of severe serum 25(OH)D deficiency in patients with FL and HL [30]. It was hypothesized that patients with insufficient vitamin D could have had impaired antitumor macrophage activity affecting their prognosis. Higher serum 25(OH)D levels obtained from exposure to ultraviolet light from sunlight might be a protective factor for the development of HL, although further investigation is needed to determine whether the aggressiveness of lymphoproliferative disease influences serum 25(OH)D levels.

Although serum vitamin D concentration in FL was investigated in two clinical studies, different cutoff points were given for defining deficiency. In the cohort of advanced-stage FL, it was set at 20 ng/mL and was significant in predicting progression-free and overall survival. On the other hand, in the cohort of patients with FL in all clinical stages, including those monitored and not immediately treated, the cutoff for deficiency was 10 ng/mL [31], a significant negative factor for progression-free survival. One hypothesized biological mechanism for this finding was that patients in these cohorts with insufficient vitamin D could have had impaired antitumor macrophage activity affecting their prognosis [48]. Data from more than 7000 participants in a study assessed the risk for HL in the general population, identifying that a 20–30% reduction in risk for HL was associated with several measures of exposure to ultraviolet radiation over the lifespan [49]. Thus, it was assumed that higher serum 25(OH)D levels obtained from exposure to ultraviolet light from sunlight might be the protective factor.

In our systematic review assessing the impact of vitamin D levels on adult patients with lymphoid malignancies, there is evidence suggesting that vitamin D plays a role in enhancing antibody-mediated antitumor macrophage activity. Insufficient vitamin D levels could result in impaired antitumor macrophage and natural killer (NK) cell activity, affecting patient prognosis. Vitamin D has been shown to modulate immune responses, including the activation and function of macrophages and NK cells involved in antibody-dependent cellular cytotoxicity (ADCC) and antibody-dependent cellular phagocytosis (ADCP) [50]. Although studies highlight the potential benefits of vitamin D supplementation in patients with lymphomas, which may improve treatment efficacy and antitumor macrophage activity [51], further investigation is required to fully understand the mechanisms underlying the relationship between vitamin D levels and immune cell function in the context of lymphoid malignancies.

### 4.2. Strengths and Limitations

Nevertheless, the current systematic review has several limitations worth highlighting, such as the fact that the included studies differed in design, sample size, patient demographics, and lymphoid cancer subtypes. This heterogeneity may have contributed to inconsistencies and difficulties in synthesizing and comparing data across research, such as varying vitamin D thresholds and measuring techniques. Moreover, the follow-up periods varied considerably between studies, which may have affected the comparability of survival outcomes and the interpretation of findings, similar to the adjustments for potential confounders, such as age, gender, race, and other variables that may have influenced the relationship between vitamin D levels and patient outcomes. In addition, the review was confined to English-only papers, which may have resulted in the removal of relevant research published in other languages, such as studies from other continents, such as South America and Africa, that could have improved the existing literature by integrating various populations in a heterogenous study. Lastly, several potential studies from different parts of the world were not included due to the study selection criteria.

### 4.3. Future Work

Building on the findings of this systematic review, future research should focus on addressing the limitations and discrepancies identified in the current literature. Prospective, well-designed, and standardized studies are necessary to further explore the relationship between vitamin D levels and lymphoid malignancies, considering factors such as geographical or ethnic differences, climate, diet, and lifestyle habits. Moreover, future studies should investigate the optimal dosage and supplementation regimen for vitamin D in patients with lymphoid malignancies, taking into account the impact of immunotherapy and corticosteroid treatments. The underlying biological mechanisms linking vitamin D deficiency and impaired antitumor macrophage activity should also be a priority for future research, which may lead to the development of novel therapeutic strategies. Additionally, researchers should examine the potential protective role of ultraviolet light exposure on lymphoproliferative disease development and aggressiveness. Lastly, further investigations into the relationship between vitamin D levels and specific lymphoma subtypes, as well as the establishment of standardized vitamin D thresholds and measuring techniques, will be essential for refining patient prognosis and guiding clinical decision-making.

## 5. Conclusions

The majority of the included studies demonstrated a significant association between vitamin D levels and patient outcomes, particularly the hazard ratios for DFS and OS being significantly higher in several studies, highlighting the importance of vitamin D supplementation in the prognosis of patients with lymphoid malignancies. Moreover, the relationship between vitamin D levels and treatment response, such as the effect of rituximab and chimeric antigen receptor T-cell (CAR-T) therapy, was demonstrated to be significant, suggesting the possibility of consequent administration of vitamin D during chemotherapy. However, it is important to note that few studies reported no significant association between vitamin D levels and patient outcomes, emphasizing the need for further research to confirm these findings. In conclusion, this systematic review suggests that vitamin D insufficiency may have negative prognostic implications in patients with lymphoid malignancies, although further research, including large-scale prospective studies and clinical trials, is warranted to confirm these findings and establish guidelines for the optimal management of patients with lymphoid malignancies.

## Figures and Tables

**Table 1:** Study characteristics.

Study and Author	Country	Study Year	Study Design	Study Quality
Drake et al. [25]	USA	2010	Prospective cohort	Excellent
Schanafelt et al. [26]	USA	2011	Prospective cohort	Excellent
Tretli et al. [27]	Norway	2012	Retrospective cohort	Good
Aref et al. [28]	Egypt	2013	Prospective cohort	Fair
Bittenberg et al. [29]	Germany	2014	Prospective cohort	Good
Kelly et al. [30]	USA	2015	Prospective cohort	Excellent
Tracy et al. [31]	USA	2017	Prospective cohort	Good
Djurasinovic et al. [32]	Serbia	2018	Prospective cohort	Good
Hohaus et al. [33]	Italy	2018	Prospective cohort	Fair
Borchmann et al. [34]	Germany	2019	Prospective cohort	Excellent
Wang et al. [35]	China	2020	Retrospective cohort	Good
Xu et al. [36]	China	2020	Retrospective cohort	Good
Mao et al. [37]	China	2021	Retrospective cohort	Good
Chen et al. [38]	China	2021	Prospective cohort	Excellent
Nath et al. [39]	USA	2022	Single-center, observational	Good

**Table 2:** Summary of findings in the included studies.

Study Number	Patients (n, % Insufficient)	Age, Years (Mean/Median)	Sex (Men, %)	Cancer Type	Outcome Measure	Duration of Follow-Up (Range/Median)
Drake et al. [25]	983 (265)	62	55.0%	DLBCL-37.6%, TCL-7.1%, MCL-7.2%, FL-28.9%, post-FL-11.9%, BL-0.8%, composite NHL-1.0%, B-cell NS-6.1%	OS, DFS	34.8 months
Schanafelt et al. [26]	543 (272)	67	70.6%	CLL-100%	OS, TTT	36 months
Tretli et al. [27]	145	56	35.8%	NR	OS	72 months
Aref et al. [28]	195	57	86.6%	BCL-100%	OS	60 months
Bittenberg et al. [29]	359	NR	NR	DLBCL-100%	OS, DFS	34.5 months
Kelly et al. [30]	183	NR	54.6%	FL-100%	OS, DFS	64 months
Tracy et al. [31]	642	60	51.5%	FL-100%	OS	59 months
Djurasinovic et al. [32]	133	58	53.4%	DLBCL-52.6%, FL-15.0%, HL-16.5%, CLL-15.8%	DFS	20 months
Hohaus et al. [33]	155	65	52.3%	Aggressive BCL-100%	DFS	NR
Borchmann et al. [34]	351	32	59.8%	HL-100%	OS, DFS	156 months
Wang et al. [35]	208	58	50.0%	DLBCL-100%	OS, DFS	29 months
Xu et al. [36]	70	61	64.2%	MCL-100%	OS, DFS	25.5 months
Mao et al. [37]	93	55	68.8%	ENKTL-100%	OS, DFS	23 months
Chen et al. [38]	332	60	53.1%	DLBCL-100%	OS, DFS	34.2 months
Nath et al. [39]	111	54	NR	DLBCL-100%	CR, OS	30 months

NR, not reported; DLBCL, diffuse large B-cell lymphoma; TCL, T-cell lymphoma; BCL, B-cell lymphoma; MCL, mantle cell lymphoma; FL, follicular lymphoma; post-FL includes splenic marginal zone lymphoma (MZL), extranodal MZL, nodal MZL, and LPL, lymphoplasmacytic lymphoma; BL, Burkitt lymphoma, NHL, non-Hodgkin lymphoma, NS, not specified; CLL, chronic lymphocytic leukemia; TTT, time to treatment; ENKTL, extranodal natural killer/T-cell lymphoma; CS, complete response.

**Table 3:** Evaluation of vitamin D levels.

Study Number	Vitamin D Threshold	Measuring Method	Mean/Median Vitamin D Level	Severe Insufficiency (%)	Overall Insufficiency (%)	Optimal Level (%)
1 [25]	<25 ng/mL	LC-MS/MS	27.4 ng/mL	5.7%	44.4%	55.6%
2 [26]	<25 ng/mL	LC-MS/MS	26.6 ng/mL	NR	39.9%	60.1%
3 [27]	<20 ng/mL	RIA	NR	NR	27.5%	72.5%
4 [28]	<20 ng/mL	ELISA	NR	8.2%	32.8%	67.8%
5 [29]	<8 ng/mL	CLIA	9.2 ng/mL	54.0%	45.7%	0.3%
6 [30]	<20 ng/mL	LC-MS/MS	31.0 ng/mL	18.1%	25.0%	56.9%
7 [31]	<20 ng/mL	LC-MS/MS	NR	NR	18.7%	81.3%
8 [32]	<30 ng/mL	CLIA	13.7 ng/mL	27.8%	72.2%	0.0%
9 [33]	<30 ng/mL	LC-MS/MS	14.0 ng/mL	NR	79.0%	21.0%
10 [34]	<30 ng/mL	ELISA	30.0 ng/mL	12.5%	49.8%	37.7%
11 [35]	<20 ng/mL	ECL	16.4 ng/mL	NR	68.3%	31.7%
12 [36]	<20 ng/mL	ECL	19.9 ng/mL	NR	57.1%	32.9%
13 [37]	<20 ng/mL	ECL	17.7 ng/mL	NR	59.1%	30.9%
14 [38]	<30 ng/mL	LC-MS/MS	16.0 ng/mL	33.1%	92.8%	7.2%
15 [39]	<30 ng/mL	LC-MS/MS	24.0 ng/mL	NR	66.0%	34.0%

NR, not reported; LC-MS/MS, liquid chromatography–tandem mass spectrometry; CLIA, chemiluminescence immunoassay; severe insufficiency, lower than 10 ng/mL; mild/moderate insufficiency, 10–24 ng/mL; RIA, radioimmunoassay; ELISA, enzyme-linked immunosorbent assay; CLIA, chemiluminescence immunoassay; ECL, electrochemiluminescence.

**Table 4:** Outcomes evaluation.

Study Number	Deaths	DFS/OS (%/HR/OR) *	Particularities
Drake et al. [25]	193 (19.6%) overall; 168 (17.1%) from lymphoid cancer	DFS-1.41, OS-1.99	Association significant only for DLBCL and TCL
Schanafelt et al. [26]	96 (17.7%) overall	TTT-1.47, OS-NS	Association significant only for TTT
Tretli et al. [27]	75 (51.7%) of lymphoma	OS-3.03	Survival among patients with the highest vitamin D levels was significantly increased
Aref et al. [28]	239 (66.6%)	OS-5.26	Average survival 48.7 months vs. 56.8 months in the normal vitamin D group
Bittenberg et al. [29]	108 (30.1%)	OS-4.10	Vitamin D deficiency impairs the effect of rituximab
Kelly et al. [30]	19 (10.4%)	DFS-NS, OS-NS	There was no association between vitamin D deficiency and cause of death
Tracy et al. [31]	78 (12.1%)	OS-2.35	Association of vitamin D insufficiency with early clinical failure
Djurasinovic et al. [32]	NR	DFS-2.92	The prevalence of 25(OH)D deficiency in the analyzed group of patients with lymphoid malignancies is high and greater in malnourished individuals
Hohaus et al. [33]	8 (5.2%)	DFS-2.88	Vitamin D deficiency improves the effect of rituximab
Borchmann et al. [34]	NR	DFS-2.13, OS-1.82	Supplemental vitamin D improves the chemosensitivity of tumors by reducing the rate of tumor growth compared with vitamin D or chemotherapy alone
Wang et al. [35]	49 (23.6%)	DFS-2.82, OS-3.72	Strong evidence was found between 25-(OH)D and prognosis in DLBCL, and the link between vitamin D and c-Myc expression was validated
Xu et al. [36]	NR	DFS-3.17, OS-8.30	25-(OH)D deficiency was a significant negative prognostic predictor for MCL
Mao et al. [37]	NR	DFS-2.60, OS-2.93	25-(OH)D deficiency was a significant negative prognostic predictor for ENKTL
Chen et al. [38]	NR	DFS-1.58, OS-2.50	Vitamin D had a protective effect on patients with DLBCL under R-CHOP treatment
Nath et al. [39]	NR	CR-2.58, OS-2.24	Insufficient vitamin D is linked to worse clinical outcomes in CAR-T recipients

* Of patients with insufficient vitamin D levels; NR, not reported; OS, overall survival; OR, odds ratio; HR, hazard ratio; DFS, disease-free survival; DLBCL, diffuse large B-cell lymphoma; TCL, T-cell lymphoma; NS, not significant.

## Abbreviations

CAR-T, chimeric antigen receptor T-cell; OS, overall survival; DFS, disease-free survival; IU, international units; FL, follicular lymphoma; HL, Hodgkin lymphoma; IL, interleukin; NR, not reported; OR, odds ratio; HR, hazard ratio; DLBCL, diffuse large B-cell lymphoma; TCL, T-cell lymphoma; NS, not significant; LC-MS/MS, liquid chromatography–tandem mass spectrometry; CLIA, chemiluminescence immunoassay; RIA, radioimmunoassay; ELISA, enzyme-linked immunosorbent assay; CLIA, chemiluminescence immunoassay; ECL, electrochemiluminescence; BCL, B-cell lymphoma; MCL, mantle cell lymphoma; MZL, marginal zone lymphoma; LPL, lymphoplasmacytic lymphoma; BL, Burkitt lymphoma, NHL, non-Hodgkin lymphoma; CLL, chronic lymphocytic leukemia; TTT, time to treatment; ENKTL, extranodal natural killer/T-cell lymphoma; CS, complete response; PRISMA, Preferred Reporting Items for Systematic Reviews and Meta-Analyses; PROSPERO, Prospective Register of Systematic Reviews; OSF, Open Science Framework.
